# Impact of SARS‐CoV2 on youth onset type 2 diabetes new diagnoses and severity

**DOI:** 10.1111/1753-0407.13301

**Published:** 2022-08-20

**Authors:** Sean DeLacey, Jennifer Arzu, Laura Levin, Adesh Ranganna, Anita Swamy, Monica E. Bianco

**Affiliations:** ^1^ Ann & Robert H. Lurie Children's Hospital of Chicago Chicago Illinois USA; ^2^ Department of Pediatrics Northwestern University Feinberg School of Medicine Chicago Illinois USA; ^3^ Department of Preventive Medicine Northwestern University Feinberg School of Medicine Chicago Illinois USA; ^4^ Northwestern University Feinberg School of Medicine Chicago Illinois USA

**Keywords:** COVID‐19, pandemic, pediatric, type 2 diabetes, 新型冠状病毒肺炎, 2型糖尿病, 儿科, 大流行

## Abstract

**Introduction:**

Initial reports show an increase in youth onset type 2 diabetes during the COVID‐19 pandemic. We aim to expand on existing evidence by analyzing trends over a longer period.

**Objectives:**

Our study aims to describe change in the amount, severity, and demographics of youth onset type 2 diabetes diagnoses during the COVID‐19 pandemic compared to the five years before.

**Methods:**

We performed a retrospective cross‐sectional review of youth (age ≤ 21) diagnosed with type 2 diabetes during the COVID‐19 pandemic (1 May 2020–30 April 2021) and the five years before (1 May 2015–30 April 2020) at a tertiary care center. Children were identified by International Classification of Diseases codes. Charts were reviewed to confirm diagnosis. Chi‐square, *t* tests, and Fisher's exact tests were used for analyses.

**Results:**

In the prepandemic era annual diagnoses of type 2 diabetes ranged from 41–69 (mean = 54.2), whereas during the pandemic period 159 children were diagnosed, an increase of 293%. The increase resulted in a higher incidence rate ratio during the pandemic than before, 2.77 versus 1.07 (*p* = .006). New diagnoses increased most, by 490%, in Non‐Hispanic Black patients. The average HbA_1c_ at presentation was higher during the pandemic (9.5% ± 2.6) (79.9 mmol/mol ± 28.2) than before (8.7%±2.1) (72.1 mmol/mol ± 23.1) (*p* = .003). Of those diagnosed during the pandemic, 59% were tested for COVID‐19 and three tested positive.

**Conclusions:**

New diagnoses of type 2 diabetes increased during the pandemic, most notably in Non‐Hispanic Black youth. There was not a significant correlation found with clinical or biochemical COVID‐19 infection in those tested.

## Graphical abstract

**Highlights**

- There has been an increase in new cases of youth onset type 2 diabetes during the pandemic.
- Non‐Hispanic Black youth have been most affected the increase in youth onset type 2 diabetes during the pandemic.
- No significant correlation was identified with clinical or biochemical COVID‐19 infection in those tested.

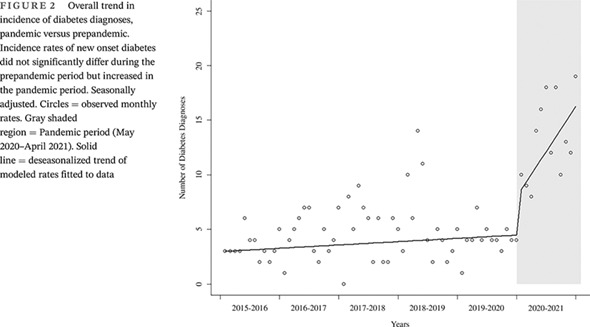

## 摘要

**背景:**

初步报告显示, 在新型冠状病毒肺炎大流行期间, 青年发病的2型糖尿病患者有所增加。我们的目标是通过分析更长时期的趋势来扩大现有证据。

**方法:**

我们对在新型冠状病毒肺炎大流行期间(2020年5月1日至2021年4月30日)和之前五年(2015年5月1日至2020年4月30日)在三级护理中心被诊断为2型糖尿病的年轻人(年龄≤21岁)进行了横向回顾。儿童通过ICD(国际疾病分类)代码进行识别。通过图表以确认诊断。分析方式采用了卡方检验, t检验和Fisher‘s精确检验。

**结果:**

在大流行前, 2型糖尿病的年诊断率为41‐69例(平均为54.2例), 而在大流行期间, 有159名儿童确诊, 增加了293%。这一增加导致大流行期间的发病率比以前更高, 分别为2.77vs1.07(p=0.006)。在非西班牙裔黑人患者中, 新诊断增加最多, 增长了490%。发病时HbA1c平均值(9.5%±2.6)(79.9mmol/mol ± 28.2)高于发病前(8.7%±2.1)(72.1mmol/mol ± 23.1)(p=0.003)。在疫情期间确诊的患者中, 59%的人进行了新型冠状病毒肺炎检测, 3人检测呈阳性。

**结论:**

在大流行期间, 新诊断的2型糖尿病增加。最显著的是在非西班牙裔黑人青年中。在接受测试的人中, 没有发现与临床或生化指标与新型冠状病毒肺炎感染有显著相关性。

## 1 INTRODUCTION

Youth onset type 2 diabetes has increased in prevalence as rates of childhood obesity have risen within the United States.^1^ Prior to the COVID‐19 pandemic, each year in the United States approximately 5000 children were newly diagnosed with type 2 diabetes and nearly one in five adolescents aged 12–18 years had prediabetes.^1^, ^2^

As of December 2021, COVID‐19 has infected over 50 million Americans and caused approximately 800 000 deaths.^3^ However, the health impact of COVID‐19 goes far beyond the infection with COVID‐19 and its associated complications. COVID‐19 changed the day‐to‐day lives of children by disrupting school attendance, altering established patterns of activity, increasing levels of familial stress, and changing dietary habits. These changes have resulted in unintended health consequences secondary to the pandemic.^4^, ^5^, ^6^

Research in COVID‐19 and childhood diabetes first focused primarily on type 1 diabetes and showed mixed results. Whereas some centers show an increase in type 1 diabetes diagnoses during the pandemic, others have not had the same findings.^7^, ^8^, ^9^, ^10^ However, given the different pathogenesis of type 1 and type 2 diabetes, these findings are not easy to extrapolate. In addition to the fact that type 2 diabetes incidence is more likely affected by changes to lifestyle, like those brought on by the COVID‐19 pandemic, it disproportionately affects racial and ethnic minority populations.^11^ Indeed, from 2002–2015 the rate of newly diagnosed cases of type 2 diabetes in children increased by 3.1% in Hispanics, 8.9% in Native Americans, and 6.3% in non‐Hispanic blacks while it rose only 0.6% in white children age 10–19 years old.^1^

Reports from other centers do indeed show a rise in cases of type 2 diabetes among youth but show variable results in terms of severity and are focused on the early pandemic period.^12^, ^13^, ^14^, ^15^, ^16^, ^17^ The cause of the rise in type 2 diabetes is likely multifaceted. SARS‐COV2 is hypothesized to directly increase the risk of hyperglycemia by acting at the angiotensin‐converting enzyme 2 receptors expressed in key metabolic organs and tissues, including pancreatic beta cells.^18^, ^19^ Adult data show an increase in fasting hyperglycemia and acute onset diabetes in those with COVID‐19 pneumonia.^20^ However, those data have not been replicated in pediatrics or tied to an increase in diagnoses of diabetes. An increase in type 2 diabetes incidence may also be anticipated because of decreased cardiovascular exercise and increased weight gain. Indeed the rate of body mass index (BMI) increase among children approximately doubled during the pandemic period compared to the prepandemic period.^6^

COVID‐19's impact on the rate of type 2 diabetes in youth is particularly important, as adult data have shown that type 2 diabetes is associated with more severe COVID‐19 outcomes.^20^, ^21^, ^22^ Therefore, if COVID‐19 is increasing rates of type 2 diabetes among youth, it is also likely increasing the risk of severe COVID‐19 infection in this population. Given the disproportionate impact of type 2 diabetes on minority populations it is also likely widening disparities in healthcare outcomes both broadly and in relation to COVID‐19.

We hypothesized that rates of diagnosis of new type 2 diabetes in youth increased during the COVID‐19 pandemic and that they increased disproportionately in ethnic and racial minorities. To test this hypothesis, we reviewed records from the 12 months after the start of the COVID‐19 pandemic in the area of our tertiary care center as well as the 5 preceding years. Between the two groups we compared the number of new diagnoses, the rise in new diagnoses, indicators of disease severity, and sociodemographic profiles.

## 2 RESEARCH DESIGN AND METHODS

We conducted a retrospective study of patients with type 2 diabetes seen within the Ann & Robert H. Lurie Children's Hospital of Chicago system diagnosed with diabetes as defined by a glycated hemoglobin (HbA_1c_) greater than or equal to 6.5% (48 mmol/mol), OR oral glucose tolerance test results (fasting >126 OR 2 h > than 200), OR a random blood glucose of >200 with consistent symptomatology from 5/1/2015 to 4/30/2021. Clinical data were extracted from electronic medical records (EMR). This study was conducted in accordance with the Declaration of Helsinki and was approved by the Lurie Children's Hospital Institutional Review Board prior to any data collection.

All patients seen at Lurie Children's Hospital or any of its satellite locations with a clinical diagnosis of type 2 diabetes were eligible for inclusion if data from their initial diagnosis encounter were available for review and occurred within the study period. Records were initially screened for by using the following International Classification of Diseases (ICD) codes for diabetes (ICD‐9 code 250.00, or ICD‐10 codes E13.9 or E11.9), if they had an encounter 5/1/2015–4/30/21, and were <21 years old on 5/1/2015. This query produced 1025 patients. The charts were screened by the research team and validated by two members of the research team (S.D. and M.B.) to confirm the clinical diagnosis of type 2 diabetes and ensure they did not meet any exclusion criteria. Exclusion criteria included: diagnosis of other forms of diabetes mellitus (such as type 1 diabetes, medication associated diabetes, cystic fibrosis‐related diabetes, or maturity onset diabetes of the young), >1 diabetes autoantibody positive, complex medical history potentially contributing to diabetes, or a BMI < 85% for age and sex. 1025 charts were reviewed and ultimately 430 patients were included for analysis. The 595 patients identified by ICD code were excluded for the following reasons: 271 with other forms of diabetes diagnoses, 206 diagnosed before study period or older than 21 at time of diagnosis, 53 miscoded, 47 with incomplete information from time of diagnosis, 15 with other complicating medical diagnoses, and 3 with BMI <85 percentile.

The period of 1 May 2015–30 April 2020 was defined as the prepandemic era and 1 May 2020–30 April 2021 was defined as the pandemic era. An emergency shelter‐in‐place order started in Chicago on 21 March 2020 and was eventually extended to 29 May 2020.^23^ Chicago public schools stopped in person teaching initially on 17 March 2020 and remained closed through the end of the 2019–2020 school year.^24^ Part of the 2020 fall quarter was fully virtual and ultimately had a hybrid learning model through the end of the 2020–2021 school year.^24^ Cases initially peaked in the spring in Illinois on May 2020, then again in November 2020 and April 2021.^25^ We believe this 12‐month time period helps capture the effects of most of the pandemic effectively.

### 2.1 Variables collected

All laboratory data were included if they were available on the EMR either through the hospital's laboratory, scanned laboratory information, or CareEverywhere. Laboratory test values from clinical laboratories included HbA_1c_, HbA_1c_ point of care (POC), serum glucose, serum osmolality, glucose POC urine ketones, and venous blood gas pH. These labs results were taken from the earliest possible collection time on the diagnosis encounter. Diabetes autoantibody results were included no matter when they were drawn in relation to diagnosis; if more than one set of antibodies was available, the most recent values were used. If a lab value was reported as greater than or less than a certain value, that value was used in the data entry.

Some vitals were taken from other encounters within a 6‐month time frame if not available at the time of diagnosis. One patient was excluded because no height was available at any time point during care and thus BMI could not be ascertained.

BMI percentiles were calculated according to the Centers for Disease Control and Prevention standard growth charts.^26^ Sex was defined by sex assigned at birth as recorded in the EMR. Information on gender was not collected.

Racial and ethnic group information and insurance information was collected from the EMR based on the predefined categories within the EMR.

### 2.2 Statistical analysis

Patient characteristics were summarized using frequencies and percentages for categorical variables, means ± SD for normally distributed continuous variables, or medians and interquartile ranges for nonnormally distributed continuous variables. To assess differences in demographic and clinical characteristics between the prepandemic and pandemic periods, we used two‐sample *t* test and Pearson's chi‐square test for continuous measures and categorical characteristics, respectively. Fisher's exact test was used for the binary variable, osmolality (OSM) ≥330 and glucose >600 (yes/no), due to expected cell counts below five. Bivariable analyses were conducted for the overall pediatric patient population as well as separately for the Hispanic and non‐Hispanic Black patient population to assess differences within these racial and ethnic subgroups. Analyses for other race and ethnic subgroups were excluded due to small sample size.

We used segmented regression analyses of interrupted time series data to evaluate the effect of the COVID‐19 pandemic on the incidence of diabetes in the overall pediatric patient population.^27^ A Poisson distribution of counts of diabetes diagnoses was assumed because no evidence of overdispersion was found (dispersion parameter = 1.25, *p* = .172). Because of the seasonal variation detected in the observed count of diabetes diagnoses, Poisson regression models were adjusted for seasonal effect by including Fourier terms consisting of two sine/cosine pairs. Durbin‐Watson test was used to assess residual autocorrelation. No evidence of autocorrelation was found after adjusting for seasonality (Durbin‐Watson = 2.17, *p* = .487). Further analysis was performed to assess whether changes in the rate of diabetes diagnoses per year differed between the Hispanic and non‐Hispanic Black patient population. Statistical analyses were conducted in R version 4.1.0 within RStudio version 1.4.1717. A two‐sided type I error rate of 0.05 was used to assess statistical significance.

## 3 RESULTS

**FIGURE 1:**
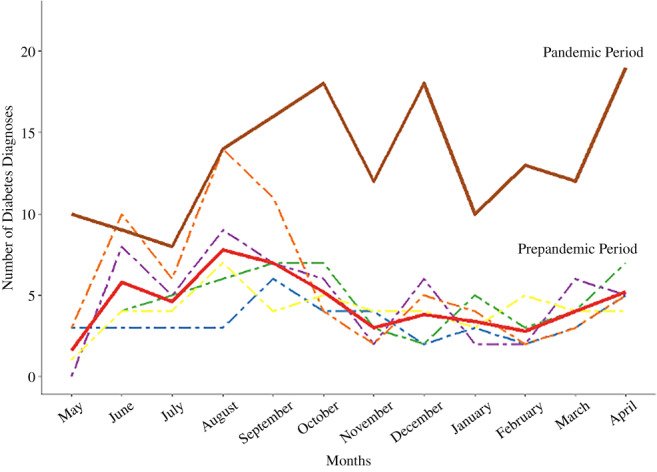
Observed monthly number of diabetes diagnoses by year, May 2015–April 2021. The number of new diagnoses of type 2 diabetes varied by month, but in each individual month of the pandemic new diagnoses of type 2 diabetes were higher than during the corresponding month during the average prepandemic period

The number of patients diagnosed with diabetes increased from the prepandemic era (1 May 2015–430 April 2020) to the pandemic era (1 May 2020–30 April 2021) (Figure 1). In the prepandemic era, a mean of 54.2 patients were diagnosed with type 2 diabetes annually (SD 10.4). During the pandemic 159 patients were diagnosed with type 2 diabetes, a 293% increase from the prepandemic mean. (Table 1).

**TABLE 1:** Differences in characteristics, prepandemic and pandemic

	Prepandemic (1 May 2015–30 April 2020), N = 271^a^	Pandemic (1 May 2020–30 April 2021), N = 159	*p* value^b^
Male sex	124 (45.76%)	86 (54.09%)	.095
Age (years)	14.1 (2.3)	14.1 (2.1)	.750
Race and ethnicity			.037*
Hispanic	170 (62.73%)	84 (52.83%)	
Non‐Hispanic Asian	11 (4.06%)	4 (2.52%)	
Non‐Hispanic Black	51 (18.82%)	50 (31.45%)	
Non‐Hispanic White	22 (8.12%)	9 (5.66%)	
Other	17 (6.27%)	12 (7.55%)	
Medicaid payor	226 (83.39%)	135 (84.91%)	.680
Initial treatment location			.345
Inpatient‐ICU	11 (4.06%)	10 (6.29%)	
Inpatient‐floor	118 (43.54%)	78 (49.06%)	
Outpatient	135 (49.82%)	69 (43.40%)	
ED and discharged	7 (2.58%)	2 (1.26%)	
HbA1C (%)	8.7 (2.1)	9.5 (2.6)	.003*
HbA1C (mmol/mol)	72.1 (23.1)	79.9 (28.2)	.003*
HbA1C ≥ 9.0%	109 (40.22%)	78 (49.37%)	.065
OSM ≥330 mOsm/kg and glucose >600 mg/dL	5 (1.85%)	1 (0.63%)	.420
BMI (kg/m^b^)	35.9 (8.0)	37.4 (8.2)	.071
BMI z‐score	2.4 (0.4)	2.4 (0.4)	0.130
BMI (%)	99.1 (98.3, 99.6)	99.3 (98.5, 99.7)	0.138
pH <7.3	23 (8.49%)	10 (6.29%)	0.409
COVID			
Negative	5 (1.85%)	86 (54.09%)	
Positive	0 (0.00%)	3 (1.89%)	
Not tested	266 (98.15%)	70 (44.03%)	

^a^ Continuous variables are expressed as mean and standard deviation except for BMI percentile, which is expressed as a median and interquartile range. Categorical variables are expressed as an “n” and percentage of the total population.

^b^ Pearson's chi‐square test; two‐sample *t* test; Fisher's exact test; Wilcoxon rank‐sum test. Hypothesis testing compare differences in characteristics prepandemic and pandemic. *p*values <.05 are marked with an asterisk (*).

Abbreviations: BMI, body mass index; ED, emergency department; HbA1C, glycated hemoglobin; ICU, intensive care unit; OSM, osmolality.

**FIGURE 2:**
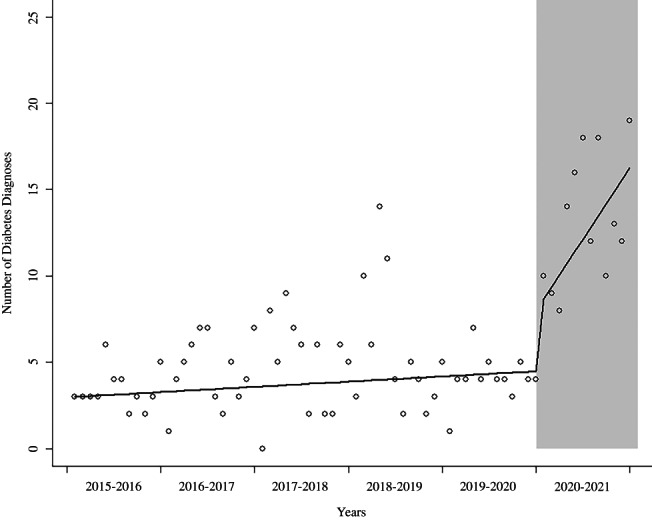
Overall trend in incidence of diabetes diagnoses, pandemic versus prepandemic. Incidence rates of new onset diabetes did not significantly differ during the prepandemic period but increased in the pandemic period. Seasonally adjusted. Circles = observed monthly rates. Gray shaded region = Pandemic period (May 2020–April 2021). Solid line = deseasonalized trend of modeled rates fitted to data

The rate of change in the number of diabetes diagnoses per year increased during the pandemic (*p* = .003). The rate of change in the number of diabetes diagnoses per year was 1.07 (95% confidence interval [CI]: 0.98, 1.16) for the prepandemic period and 2.77 (95% CI: 1.40, 5.47) during the pandemic (Table 2, Figure 2).

**TABLE 2:** Interrupted time series model–overall

	IRR	95% CI	*p* value^a^
(Intercept)	4.81	4.06, 5.64	**<.001**
Prepandemic trend	1.07	0.98, 1.16	.122
Onset of pandemic	0.56	0.19, 1.64	.292
Pandemic trend	2.77	1.40, 5.47	**.006**

Abbreviations: CI, confidence interval; IRR, incidence rate ratio.

^a^ *p* values are two sided. Results have been adjusted for seasonality.

The increase in diagnoses was not evenly distributed among racial and ethnic groups. New diagnoses increased 490% in Non‐Hispanic Black patients, 247% in Hispanic patients, and 196% in Non‐Hispanic White patients. Hispanic patients constituted most type 2 diabetes diagnoses both during and before the pandemic (62.7% vs. 52.8%). However, the proportion of patients identifying as Non‐Hispanic Black grew during the pandemic from 18.82% to 31.45%. Non‐Hispanic White patients represented a small proportion of the population both during and before the pandemic (Table 1).

No statistically significant difference was found in the rate of change in the number of diabetes diagnoses per year prepandemic for non‐Hispanic Black patients compared to Hispanic patients (*p* = .908). Before the pandemic, the rate of change in the number of diabetes diagnoses per year were 1.09 (95% CI: 0.98, 1.21) for Hispanic patients and 1.10 (95% CI: 0.61, 2.18) for non‐Hispanic Black patients. The increase in the number of diabetes diagnoses at the beginning of the pandemic compared to the end of the prepandemic period for non‐Hispanic Black patients (incident rate ratio [IRR]: 3.70; 95% CI: 1.61, 8.50) significantly differed from Hispanic patients (IRR: 1.16; 95% CI: 0.61, 2.18) (*p* = .021). During the pandemic, the rate of change in the number of diabetes diagnoses per year for non‐Hispanic Black patients was not statistically different from Hispanic patients (*p* = .165). For Hispanic patients, the rate was 2.76 (95% CI: 1.14, 6.72) and for non‐Hispanic Black patients, it was 1.11 (95% CI: 0.37, 3.35) (Table 2).

There was no significant difference in patients diagnosed with type 2 diabetes who were publicly insured before and during the pandemic (82.3% vs. 82.4%) (*p* = .979). Although females represented the majority of those diagnosed with type 2 diabetes before the pandemic (54.2%), males represented the majority during the pandemic (54.1%). The difference in sex proportion approached significance (*p* = .095) (Table 1).

In general, prevalence of acidosis and hyperosmolarity at diagnosis represented a small part of the population. Prevalence of both events was similar during the pandemic and before the pandemic among those with a new diagnosis of type 2 diabetes. The percentage of new diagnoses with a pH <7.30 prepandemic was 8.5% and was 6.3% during the pandemic (*p* = .409). Rates of acidosis were too small to differentiate significant differences between racial and ethnic subgroups.

Diabetes was more advanced at diagnosis during the pandemic than prior. The average HbA_1c_ at presentation was higher during the pandemic (9.5% ± 2.6) (79.9 mmol/mol ± 28.2) than prior (8.7% ±2.1) (72.1 mmol/mol ± 23.1) (*p* = .003).

A total of 55.9% of patients diagnosed during the pandemic period were tested for COVID‐19 either at diagnosis or within the month of being diagnosed; of those only three patients were positive for COVID‐19 (Table 1)

## 4 DISCUSSION

Our findings demonstrate an increase in new diagnoses of type 2 diabetes during the COVID‐19 pandemic, which is consistent with the existing literature on the subject.^12^, ^15^, ^16^ Like other centers our rates of type 2 diabetes increased, though ours appears to be a more dramatic increase than previously described. The more significant rise may be related to a longer period of study during the pandemic.

Some studies have reported an increased rate of diabetic ketoacidosis (DKA) and diabetes‐related admissions during the pandemic both for those with type 1 and type 2 diabetes.^7^, ^12^, ^14^, ^16^ Our findings may differ for a variety of reasons. Althoush some studied the difference in total number of DKA or hyperglycemic hyperosmolar state events, we looked at the proportion of new diagnoses that presented with acidosis or hyperosmolarity. Second, our findings compared rates to the 5 years before and not just the preceding year, giving a better powered comparison. And finally, we assessed a later part of the pandemic, when families may have been more comfortable accessing care. As a result, although the absolute number of admissions for acidosis or hyperosmolarity increased, it represented a similar proportion of those children diagnosed with diabetes.

Limited information is available on concurrent diabetes diagnosis and COVID‐19 diagnosis particularly in pediatrics. We had three patients with positive COVID‐19 testing at time of diagnosis, only one of whom had clinically significant infection. This is consistent with other studies reporting that COVID‐19 positivity does not appear to correlate with diagnosis.^13^ However, it is difficult to know whether COVID‐19 infection occurred prior to a patient's presentation and ultimately increased their risk of diabetes. We did not collect information on prior infection. We also do not have COVID‐19 results on a large proportion of patients because COVID‐19 testing was done routinely only on those admitted for inpatient diabetes education or treatment. Overall, in our population there was insufficient information to establish or refute any direct connection between COVID‐19 infection and diabetes onset.

One possible explanation of the increased rates of type 2 diabetes is the concurrent increase in BMI and obesity in the pediatric population during COVID‐19.^6^ Indeed, obesity is a known risk factor for type 2 diabetes. We did not find a statistically significant difference in the markers of obesity (BMI, BMI percentile, and BMI z‐score) in our population before and after the pandemic (Table 1). However, the lack of difference has a variety of possible explanations including a potential weight loss prior to diagnosis in those with more advanced hyperglycemia at presentation.

Indeed, the pandemic affected children's lives in many ways aside from direct infection. Among them were school closings, discontinuance of sports/afterschool activities, increased parental, and illness among family members. Although school closings helped prevent disease spread, they likely also had a large impact on the metabolic health of students. Indeed prior to the pandemic, studies documented a tendency for children's BMI to increase during the summer months when they are out of their structured school environment.^28^, ^29^ For those with limited access to food and activity outside of school that impact could be more profound.^30^, ^31^, ^32^ BMI increased during the pandemic as did sedentary behavior.^33^ Sedentary behavior, independent of weight status, can decrease insulin sensitivity and predispose to diabetes.^34^

### 4.1 Strengths and limitations

A strength of our study is the large racially and ethnically diverse patient population. Our study also benefits from a long period of study before the start of the pandemic and collection of data far into the pandemic when the effects of behavior changes were more likely to be seen. The extension of our concept of the pandemic era into the first half of 2021 may better capture the increased rates of type 2 diabetes as social changes that affected weight status and nutrition likely had cumulative effects. Another strength of our study is the relatively large proportion of our patients with a COVID‐19 test done at diagnosis compared to previously published information.

One clear limitation of our study is that we cannot fully quantify incidence of type 2 diabetes as our sample is from only one tertiary care center. We do not have an accurate denominator to calculate incidence. We do know from facility estimates that new patient encounters within the endocrine department increased approximately 112% during our specified pandemic period compared to the 2 years before. Despite the increase it is not proportionate to the increase seen within the new diagnoses of type 2 diabetes (as noted previously an increase of 293%).

Other limitations of our study are primarily related to its retrospective nature. The lab data were collected from the EMR exclusively, but lab values were drawn from a variety of different labs with nonstandardized values. The racial and ethnic data were derived from the medical record and thus may not be compatible with patient self‐identified racial or ethnic identify and do not include potentially relevant other racial groups or subgroups. The population of Asian, American Indian, and Pacific Islander populations was not large enough to make any statistically significant conclusions.

There was a variety of information that was not available to help elucidate the etiology of the increase in diagnoses. Other information that may have been helpful was data on school attendance, familial illness, parental job status, food security, primary care access, and trends in weight in the preceding months. As social determinants of health become more standard in medical documentation, our understanding of widespread disease trends will increase.

## 5 CONCLUSIONS

Type 2 diabetes diagnoses increased in the pediatric population during the COVID‐19 pandemic. The increase was more pronounced in some racial and ethnic subgroups, particularly in the Non‐Hispanic Black population. Diabetes was more advanced at diagnosis as represented by a higher average HbA_1c_ but did not manifest in a higher proportion of cases of acidosis or hyperosmolar hyperglycemia in our population. Diabetes showed no relationship with concurrent clinical or biochemical COVID‐19 diagnosis in our population, though testing was not universal. Other possible explanations for the rise are changes in the community that negatively affected nutrition, access to primary health care, exercise opportunities, and transportation. The hypothesis is supported by the disproportionate increase in type 2 diabetes among Hispanic and non‐Hispanic Black populations who traditionally experience more of these health barriers.

## AUTHOR CONTRIBUTIONS

S.D. and M.B. conceived the study concept and design. All authors participated in validating the data and took part in interpretation of the results. S.D. primarily was responsible for preparing the manuscript. J.A. primarily was responsible for the statistical modeling. All authors participated in the decisions to submit the publication and reviewed the final manuscript. M.B. is the guarantor of this work, and as such, had full access to all of the data and takes responsibility of the integrity of the data and the accuracy of the data analysis.

## FUNDING INFORMATION

NUCATS services and support through grant UL1TR001422 (REDCap and BCC support). S.D.'s work is supported by Ruth L. Kirschstein National Research Service Award T32 DK007169 from the National Institute of Diabetes and Digestive and Kidney Diseases (NIDDK). M.B. is supported by NIH 3R01DK118403‐02S1 from NIDDK.

## CONFLICT OF INTEREST

A.S. is a speaker for Novo Nordisk, Dexcom, and Insulet. All other authors do not have conflict of interest to disclose.
